# Distinct Microbial Signatures between Periodontal Profile Classes

**DOI:** 10.1177/00220345211009767

**Published:** 2021-04-27

**Authors:** J.T. Marchesan, K. Moss, T. Morelli, F.R. Teles, K. Divaris, M. Styner, A.A. Ribeiro, J. Webster-Cyriaque, J. Beck

**Affiliations:** 1 Division of Comprehensive Oral Health, Adams School of Dentistry, University of North Carolina at Chapel Hill, Chapel Hill, NC, USA; 2 Division of Oral and Craniofacial Health Sciences, Adams School of Dentistry, University of North Carolina at Chapel Hill, Chapel Hill, NC, USA; 3 Department of Basic and Translational Sciences, University of Pennsylvania, School of Dental Medicine, Philadelphia, PA, USA; 4 Division of Pediatric and Public Health, Adams School of Dentistry, University of North Carolina at Chapel Hill, Chapel Hill, NC, USA; 5 Department of Epidemiology, Gillings School of Global Public Health, University of North Carolina at Chapel Hill, Chapel Hill, NC, USA; 6 Department of Medicine, Division of Endocrinology and Metabolism, University of North Carolina at Chapel Hill, Chapel Hill, NC, USA; 7 Division of Diagnostic Sciences, Adams School of Dentistry, University of North Carolina at Chapel Hill, Chapel Hill, NC, USA; 8 Division of Oral and Craniofacial Health Sciences, Adams School of Dentistry, University of North Carolina at Chapel Hill, Chapel Hill, NC, USA

**Keywords:** dysbiosis, biofilm, adaptive immunity, periodontitis, sex characteristics, race factors

## Abstract

Precise classification of periodontal disease has been the objective of concerted efforts and has led to the introduction of new consensus-based and data-driven classifications. The purpose of this study was to characterize the microbiological signatures of a latent class analysis (LCA)–derived periodontal stratification system, the Periodontal Profile Class (PPC) taxonomy. We used demographic, microbial (subgingival biofilm composition), and immunological data (serum IgG antibody levels, obtained with checkerboard immunoblotting technique) for 1,450 adult participants of the Dental Atherosclerosis Risk in Communities (ARIC) study, with already generated PPC classifications. Analyses relied on *t* tests and generalized linear models with Bonferroni correction. Men and African Americans had higher systemic antibody levels against most microorganisms compared to women and Caucasians (*P* < 0.05). Healthy individuals (PPC-I) had low levels of biofilm bacteria and serum IgG levels against most periodontal pathogens (*P* < 0.05). Subjects with mild to moderate disease (PPC-II to PPC-III) showed mild/moderate colonization of multiple biofilm pathogens. Individuals with severe disease (PPC-IV) had moderate/high levels of biofilm pathogens and antibody levels for orange/red complexes. High gingival index individuals (PPC-V) showed moderate/high levels of biofilm *Campylobacter rectus* and *Aggregatibacter actinomycetemcomitans*. Biofilm composition in individuals with reduced periodontium (PPC-VI) was similar to health but showed moderate to high antibody responses. Those with severe tooth loss (PPC-VII) had significantly high levels of multiple biofilm pathogens, while the systemic antibody response to these microorganisms was comparable to health. The results support a biologic basis for elevated risk for periodontal disease in men and African Americans. Periodontally healthy individuals showed a low biofilm pathogen and low systemic antibody burden. In the presence of PPC disease, a microbial-host imbalance characterized by higher microbial biofilm colonization and/or systemic IgG responses was identified. These results support the notion that subgroups identified by the PPC system present distinct microbial profiles and may be useful in designing future precise biological treatment interventions.

## Introduction

Periodontitis is a condition characterized by an exaggerated inflammatory response associated with dysbiotic ecological changes in a healthy commensal oral microbiome. Whole microbial community changes take place as disease develops, with high levels of traditional periodontal microorganisms previously coined “red” and “orange” complex consistently identified in periodontitis sites (Socransky et al. 1998; Haffajee et al. 2009; Teles et al. 2012). Still, technological advancements revealed that the microbiome is a tangled, complex system of interactions with no specific microbial model that currently explains the onset and progression of disease entities (Teles et al. 2021). This microbiome complexity and diversity agrees with the historic evidence that clinical periodontitis is heterogeneous by nature.

As knowledge in health and disease pathogenesis expands, new classification systems are developed to better represent disease subphenotypes with similar and overlapping clinical presentations (Offenbacher et al. 2016). Recently, multidimensional profiles that combine clinical and biological features to define periodontal traits have been proposed. In addition to the traditional clinical and radiographic variables, known risk factors that affect the oral microbiome and host response (smoking and diabetes) were included in the current 2017 World Workshop (WW17) disease classification (Tonetti et al. 2018). Others have used direct measurements of biological variables (i.e., levels of proinflammatory interleukin [IL]–1β and periodontal pathogens) to define distinct oral traits (Offenbacher et al. 2016; Agler et al. 2019). These efforts that directly and indirectly address the biological aspects of disease may help uncover categories that better represent periodontitis complexity.

Precision medicine is an emerging approach in health care, wherein, rather than providing a traditional, one-size-fits-all treatment based on the expected response of an average patient, it proposes to stratify each patient into disease subclasses with a common biological basis (Divaris 2019). Knowledge on which biochemical or physiological processes cause the issue will give health providers the ability to precisely diagnose, prevent, and identify a therapy that matches alike individuals.

To enable such applications, latent class analysis (LCA) is a suitable data-driven statistical modeling technique that has been used to identify precise care in other fields (Calfee et al. 2014; Ghanbari et al. 2018; Ahanchi et al. 2019; Petersen et al. 2019). This approach has been recently applied to periodontal disease, leading to the identification of 7 homogeneous clinical phenotypes named periodontal profile classes (PPCs) (Morelli et al. 2017). This validated stratification method has been previously applied to >14,500 individuals to address disease comorbidities, genetics, and oral health behavioral questions (Beck et al. 2018a, 2018b; Marchesan et al. 2018; Morelli et al. 2018; Sen et al. 2018; Agler et al. 2019; Marchesan, Byrd, et al. 2020; Marchesan, Girnary, et al. 2020; Morelli et al. 2020). From a biological standpoint, PPCs are strongly associated with local (GCF-IL-1β), systemic inflammation (serum IL-6 and C-reactive protein [CRP]) (Beck et al. 2018b). Together, the evidence suggests that the periodontal classes may also reflect 7 phenotypes or subphenotypes with distinct patterns of microbial dysbiosis and immune responses.

In this article, we provide the first microbial and systemic antibody characterization of the 7 periodontal phenotypes. The selection of “orange” and “red complex” subgingival microorganisms (Socransky et al. 1998) will provide a foundational microbial signature for each subphenotype. We hypothesized that these periodontal classes (phenotypes) would be significantly associated with higher levels of subgingival periodontal pathogens and/or higher systemic antibody levels compared to PPC-I (health).

## Methods

### Study Population and Disease Classification

The Atherosclerosis Risk in Communities (ARIC) study is a prospective investigation of the development of atherosclerosis/cardiovascular disease in 15,792 American residents. The current study consisted of a random subsample (*n* = 1,450) of the Dental-ARIC (DARIC) study, an ancillary cross-sectional investigation added during visit 4 of the parent study. The study has conformed to the Strengthening the Reporting of Observational Studies in Epidemiology (STROBE) guidelines. Detailed information about the study is presented in the Appendix and has been previously described (Beck et al. 2001). All participants provided written informed consent to an institutional review board–approved protocol. Data available from all the participants were used, with few exclusions due to missing data: smoking status (*n* = 2), diabetes (*n* = 2), body mass index (BMI; *n* = 2), and dental utilization (*n* = 4). LCA was based on 1) ≥1 site with interproximal attachment level (iCAL) ≥3 mm, 2) ≥1 site with probing depth (PD) ≥4 mm, 3) bleeding on probing (BOP; percentage of sites with BOP, dichotomized at 50% or ≥3 sites/tooth), 4) gingival inflammation index (GI, dichotomized as GI = 0 vs. GI ≥1), (5) plaque index (PI; dichotomized as PI = 0 vs. Pl ≥ 1), 6) presence/absence of prosthetic crowns/teeth, and 7) tooth presence/absence.

### Bacterial Biofilm Composition and Serum IgG Antibody Analysis

Subgingival biofilm samples and serum samples were collected as previously described (Beck et al. 2001, 2005). One biofilm was used from each individual, sampling the mesiobuccal site of the right maxillary first molar. Samples were collected into tubes with sterile TE buffer and stored until further analysis. One serum sample/individual was also collected and evaluated. The samples were assayed by checkerboard immunoblotting technique (Beck et al. 2005). Periodontal microorganisms selected for the analysis were classic representatives reported to be associated with periodontal infection (Socransky et al. 1998). Levels of microorganisms were expressed as mean log_10_ counts using microbial standards for *Porphyromonas gingivalis*, *Prevotella intermedia*, *Treponema denticola*, *Tannerella forsythia*, *Campylobacter rectus*, *Fusobacterium nucleatum*, *Aggregatibacter actinomycetemcomitans*, and *Prevotella nigrescens*. Serum IgG levels were expressed as micrograms/millimeter of IgG-specific titers (log_10_), as previously described (Sakellari et al. 1997).

### Statistical Analysis

Frequency distributions, means, and standard errors were estimated. Bivariate relationships were assessed by *t* tests and general linear models, adjusted for race, age, sex, diabetes, BMI, smoking, education, and dental utilization. We used a Bonferroni multiple-testing correction. Statistical analysis was performed using SAS (Version 9.4; SAS Institute).

## Results

### Demographics

Analysis of the demographic characteristics showed that each class was distinct with significant differences identified in all parameters (Table 1). Nonparticipation was limited to participants not selected for bacterial biofilm samples (random). Appendix Table 1 shows the original definition of the PPC system (Morelli et al. 2017) with the recent reworded definition (Beck et al. 2020). PPC-I (health) included the healthiest group, mainly composed of Caucasian females, younger individuals, those with the highest number of never-smokers, mostly nondiabetics, and those with the lowest BMI, lowest percentage of individuals with basic-level education, and frequent dental visits. Individuals with mild to moderate disease (PPC-II to PPC-III) were also mainly composed of Caucasians, mostly males, and those with a high education level and frequent dental visits but had a higher percentage of smokers compared to PPC-I (health). Those with severe disease (PPC-IV) had a similar race distribution within the class, were mostly males, included ~20% of smokers, and had the highest percentage of diabetic individuals (25%) among all classes. High gingival inflammation (PPC-V) was mainly composed of African Americans, females, never smokers, nondiabetics, and those with the highest BMI, and the majority were episodic dental visitors. PPC-VI (moderate tooth loss [TL]/reduced periodontium) were mostly Caucasians, with equal sex distribution, the highest percentage of smokers (24%), and a high BMI, and they were regular dental visitors. Severe tooth loss (PPC-VII) was mainly composed of Caucasian females, former smokers, those with a high percentage of diabetes (21%), the highest number of individuals with a low level of education, and mostly infrequent dental visitors.

**Table 1.** Dental Atherosclerosis Risk in Communities Study Participants’ Demographic Information, Stratified According to the Periodontal Profile Class System (*N* = 1,450).

Characteristic	PPC-I: Health	PPC-II: Mild	PPC-III: Moderate	PPC-IV: Severe Disease	PPC-V: Mild TL/High GI	PPC-VI: Moderate TL/Reduced Periodontium	PPC-VII: Severe TL	*P* Value
Entire sample, *n* (%)	384 (27)	235 (16)	216 (15)	123 (9)	123 (9)	174 (12)	195 (13)	
Age, mean (SD), y	62 (6)	63 (6)	64 (6)	63 (6)	62 (6)	65 (6)	63 (6)	<0.0001
Race, *n* (%)								
African American	7 (2)	6 (2)	2 (1)	50 (41)	87 (71)	21 (12)	41 (21)	<0.0001
Caucasian	377 (98)	229 (98)	214 (99)	73 (59)	36 (29)	153 (88)	154 (79)	
Gender, *n* (%)								
Female	246 (64)	94 (40)	85 (39)	42 (34)	60 (49)	87 (50)	106 (54)	<0.0001
Male	138 (36)	141 (60)	131 (61)	81 (66)	63 (51)	87 (50)	89 (46)	
Smoking status, *n* (%)								
Never smoker	200 (52)	102 (43)	79 (37)	54 (44)	56 (46)	56 (34)	55 (30)	<0.0001
Former	158 (41)	115 (49)	105 (49)	43 (35)	51 (42)	84 (48)	93 (48)	
Current	26 (7)	18 (8)	32 (15)	25 (21)	15 (12)	34 (20)	47 (24)	
Diabetes mellitus, *n* (%)^a^								
Diabetic	35 (9)	31 (13)	30 (14)	31 (25)	24 (20)	29 (17)	42 (22)	<0.0001
Nondiabetic	349 (91)	204 (87)	186 (86)	92 (75)	97 (80)	145 (83)	153 (78)	
Body mass index, mean (SD), kg/m^2^	27.5 (5)	28.1 (5)	28.4 (5)	29.4 (6)	31.1 (7)	28.5 (5)	30.0 (6)	<0.0001
Education, *n* (%)^b^								
Basic	21 (6)	15 (6)	13 (6)	33 (27)	37 (30)	27 (16)	55 (28)	<0.0001
Intermediate	162 (43)	105 (45)	95 (44)	36 (30)	45 (37)	92 (53)	94 (48)	
Advanced	201 (52)	115 (49)	108 (50)	54 (43)	41 (33)	55 (32)	46 (24)	
Dental utilization (visits), *n* (%)								
Regular	349 (91)	199 (85)	184 (85)	64 (52)	54 (44)	129 (74)	82 (42)	<0.0001
Episodic	33 (9)	35 (15)	32 (15)	59 (48)	69 (56)	45 (26)	112 (58)	

Data excluded from the analysis due to missing information: smoking (*n* = 2), diabetes (*n* = 2), and dental utilization (*n* = 4). *P* values are based on general linear models.

GI, gingival inflammation index; PPC, Periodontal Profile Class; TL, tooth loss.

a Diabetes is a categorical variable determined by a questionnaire.

b Education defined as the following: basic, grade school, zero years of education, or high school with no degree; intermediate, high school graduate or vocational school; advanced, college, graduate school, or professional school.

### Clinical Parameters

Each periodontal class, or subphenotype of periodontitis, presented a distinct clinical trait (Table 2, graduated color-coded/row). PPC-I (health) is mostly represented in green, with the lowest clinical values of disease. PPC-II (mild) had moderate levels of inflammation (BOP, GI) and plaque (PI) but mild tissue destruction (iCAL). In contrast, PPC-III (moderate) showed moderate tissue destruction (iCAL ≥3 mm) but mild gingival inflammation. Still, individuals in the mild to moderate disease class had similar tooth levels of loss. Severe disease (PPC-IV) showed the highest amounts of subgingival biofilm (percent plaque index score), bleeding, iCAL ≥3 mm, and PD ≥4 mm. Individuals in PPC-V (mild TL/high GI) were marked by the highest mean extent GI score among all classes, high amounts of biofilm (percent plaque index score), and moderate tooth loss. PPC-VI (moderate TL/reduced periodontium) individuals had a healthy-like subgingival biofilm, moderate disease parameters (iCAL, PD, BOP, and GI), and lost almost half of their dentition (often a marker for significant past disease). Those with severe disease (PPC-VII) showed moderate percentages of all clinical parameters and lost the majority of their dentition. For comparison purposes, Appendix Table 2 shows the number of individuals distributed according to PPC and the WW2017 classification. In the PPC system, most individuals are considered healthy (PPC-I). In the WW17, most individuals are disease stage II, with similar distribution among stages III and IV. The distinct classes of PPC-V, PPC-VI, and PPC-VII are distributed among the 4 WW17 stages. This demonstrates that patterns of disease identified by human-defined rules are clearly distinct from data-driven phenotypes (PPC), suggesting that traditional classification systems may group different subtypes of disease into 1 disease class.

**Table 2.** Mean (SE) Adjusted Clinical Measurements Stratified by Periodontal Profile Classes.^a^

Characteristic	PPC-I: Health, *n* (%)	PPC-II: Mild, *n* (%)	PPC-III: Moderate, *n* (%)	PPC-IV: Severe Disease, *n* (%)	PPC-V: Mild TL/High GI, *n* (%)	PPC-VI: Moderate TL/Reduced Periodontium, *n* (%)	PPC-VII: Severe TL, *n* (%)	*P* Value
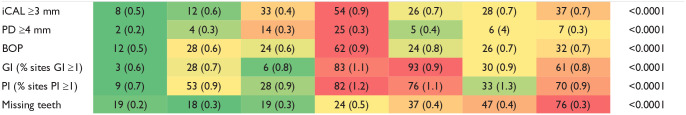								

Color-coding system applied across all periodontal classes in accordance to each clinical variable (separate coding/row): green = low, yellow = mild, orange = moderate, red = high percentages. *P* values based upon general linear models.

BOP, bleeding on probing; GI, gingival inflammation index; plaque index; iCAL, interproximal clinical attachment levels; PD, probing depth; PI, plaque index; PPC, Periodontal Profile Class; TL, tooth loss.

a Data adjusted for race/center, age, sex, diabetes, body mass index, smoking (3 levels: current smoker, former smoker, nonsmoker), education (3 levels: basic, intermediate, advanced), and dental utilization (frequent dental visits, episodic dental visits).

### Microbial Profile by Sex, Race, and Age

To better characterize this population, microbiological/host response data were stratified based on biological variables. While periodontal pathogen colonization of the biofilm was not different based on sex, the systemic antibody levels were significantly higher in men compared to women (*P* < 0.05, Fig. A). African Americans had significantly higher amounts of biofilm microorganisms and higher systemic host responses reflected in IgG levels to most microorganisms when compared to Caucasians (Fig. B). Age was less impactful, with higher systemic antibody responses to 3 periodontal pathogens identified in older individuals (Fig. C). Among the biological variables evaluated, race had the most dramatic impact on the microbial-antibody responses, followed by sex, with men presenting higher systemic antibody responses.

**Figure.**
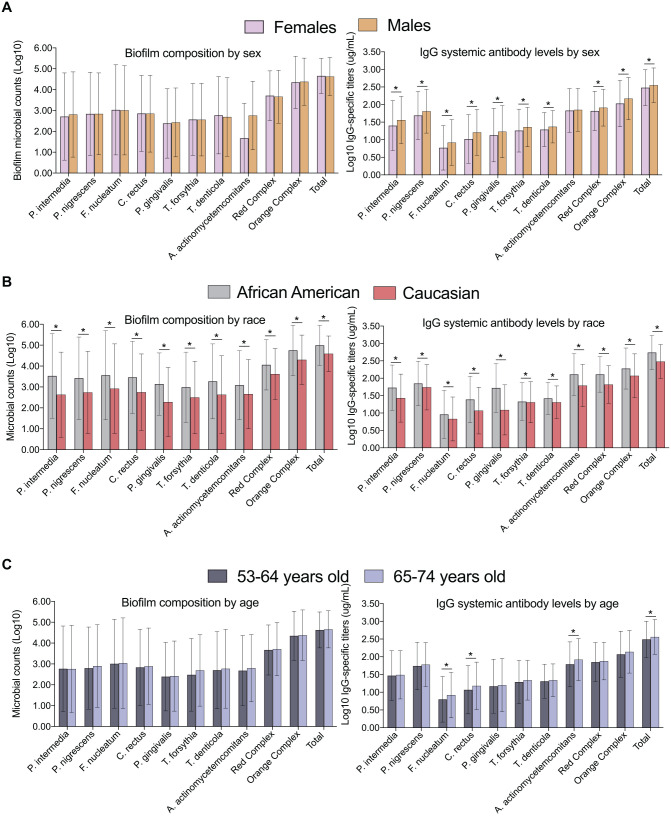
Microbial biofilm composition and serum antibody levels to 8 periodontal microorganisms in individuals stratified by (**A**) sex, (**B**) race, and (**C**) age. The bars denote means and the vertical brackets indicate SD. The asterisk (*) denotes statistically different between groups at *P* < 0.05.

### Periodontal Profile Classes and Bacterial Biofilm Composition

Among all bacteria, *P. intermedia*, *C. rectus*, *T. forsythia*, and *T. denticola* levels were significantly different among classes (after Bonferroni correction; Table 3). *P. gingivalis* and *A. actinomycetemcomitans* levels were also different among PPC classes but did not pass Bonferroni correction. Health (PPC-I) and moderate TL/reduced periodontium (PPC-VI) showed low mean counts for all biofilm microorganisms. Both mild disease (PPC-II) and moderate disease (PPC-III) had mild increases in levels of *C. rectus* and *T. forsythia*. In addition, individuals with mild disease (PPC-II), who had high amounts of subgingival biofilm, had moderate/high levels of orange complex*P. intermedia* compared to healthy controls. Severe disease (PPC-IV) was dominated by high levels of red complex *T. denticola* and a moderate increase in levels of orange complex microorganisms. High gingival inflammation (PPC-V) had moderate loads of most microorganisms and significantly higher *C. rectus* levels. Subjects with severe tooth loss (PPC-VII), a disease-associated bacterial biofilm phenotype, had significantly higher levels of 6 of the 8 microorganisms. In sum, the data showed that health (PPC-I) and reduced periodontium (PPC-VI) had low levels of classic periodontal pathogens. Mild to moderate disease (PPC-II-III) showed an “incipient” dysbiosis, while severe disease (PPC-IV), high gingival inflammation (PPC-V), and severe tooth loss (PPC-VII) presented a higher shift toward a disease-associated microbiota.

**Table 3.** Adjusted Log Microbial Counts (Mean, SE) by PPC-Stage Definition of Disease.^a^

Plaque Bacteria	PPC-I: Health	PPC-II: Mild	PPC-III: Moderate	PPC-IV: Severe Disease	PPC-V: Mild TL/High GI	PPC-VI: Moderate TL/Reduced Periodontium	PPC-VII: Severe TL	*P* Value
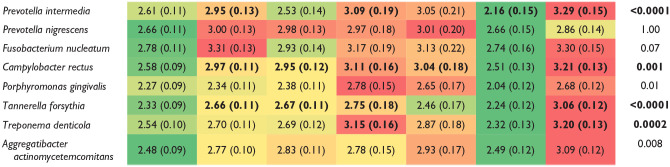								

*P* values based upon general linear models. Bold indicates significant using PPC-I as reference (Bonferroni corrected). Color-coding system applied across all periodontal classes in accordance to levels of microorganisms (separate coding/row): green = low, yellow = mild, orange = moderate, red = high levels.

GI, gingival inflammation index; PPC, Periodontal Profile Class; TL, tooth loss.

a Data adjusted for race/center, age, sex, diabetes, body mass index, smoking (3 levels: current smoker, former smoker, nonsmoker), education (3 levels: basic, intermediate, advanced), and dental utilization (frequent dental visits, episodic dental visits).

For comparison, Appendix Table 3A shows the microbial biofilm composition according to traditional Centers for Disease Control/American Academy of Periodontology (CDC/AAP) classification, and Appendix Table 3B shows the 2017 world workshop classification (WW17 modified). CDC/AAP health to mild disease demonstrated low levels of traditional periodontal pathogens (Appendix Table 3A). A mild shift toward a disease-associated phenotype was identified in individuals with CDC/AAP moderate disease. CDC/AAP severe disease showed the most disease-associated biofilm composition, with significantly high levels of *P. gingivalis* (lost significance after Bonferroni adjustment). The WW2017 classification was superior in identifying groups that were microbiologically homogeneous (see color contrast between classes by comparing Appendix Table 3A, B). WW17 stage I presented with the lowest levels of periodontal pathogens, with an incipient dysbiosis identified in stages II, III, and IV. Importantly, *F. nucleatum* was significantly higher compared to stage I (Bonferroni adjusted), with a similar trend observed for levels of *P. gingivalis* (Appendix Table 3B, *P* = 0.09). Stage I, however, had a small number of individuals identified as being WW2017 healthy in this study population.

### Periodontal Profile Class and Systemic IgG Levels

Systemic IgG responses against *P. gingivalis* and *C. rectus* were significantly higher in PPC-IV and PPC-VI when compared to healthy controls (Bonferroni adjusted; Table 4). Antibody levels against these 2 pathogens were also the highest in CDC/AAP moderate and severe disease (Appendix Table 4A). *P. gingivalis* was the only microorganism with significantly higher antibody titers in the WW17 classification (Appendix Table 4B). Of the 7 PPC classes, 5 of them (PPC-I to PPC-V) had levels of biofilm microorganisms generally similar to the systemic antibody responses (see color pattern differences between in Tables 3 and 4). Health (PPC-I) (health), which had low levels of biofilm microorganisms, was significantly associated with a low IgG response against most microorganisms. Mild to moderate disease (PPC-II to PPC-III), the classes with “incipient biofilm dysbiosis,” had mild antibody responses for most microorganisms. Severe disease (PPC-IV) and high gingival inflammation (PPC-V) had moderate to severe levels of periodontal bacteria in biofilm (Table 3) and moderate to severe systemic antibody responses against red and orange complex bacteria. The remaining 2 classes, reduced periodontium (PPC-VI) and severe tooth loss (PPC-VII), showed opposite directional changes of bacterial biofilm composition in comparison to systemic responses. Reduced periodontium (PPC-VI), characterized by a reduced periodontium and low levels of biofilm periodontal pathogens, showed moderate to high systemic IgG antibody titers against most bacteria. A low to mild systemic IgG response was seen for most microorganisms evaluated in severe disease (PPC-VII), which was the class with high amounts of a disease-associated bacteria in the biofilm (Tables 3 and 4). Among the 8 microorganisms evaluated in this study, the classic periodontal pathogen *P. gingivalis* was uniquely in higher disease states, independent of the classification system used (Appendix Table 4A, B). Together, the data show that all disease classes were associated with an imbalance between the microbial biofilm composition and the systemic acquired immune response against periodontal pathogens when compared to healthy controls.

**Table 4.** Mean (SE) Adjusted Log Microbial IgG Levels Stratified by Periodontal Profile Classes (*n* = 1,450).^a^

Plaque Bacteria	PPC-I: Health	PPC-II: Mild	PPC-III: Moderate	PPC-IV: Severe Disease	PPC-V: Mild TL/High GI	PPC-VI: Moderate TL/Reduced Periodontium	PPC-VII: Severe TL	*P* Value
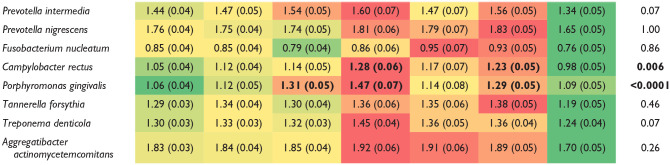								

*P* values based upon general linear models. Bold indicates significant using PPC-I as reference (Bonferroni corrected). Color-coding system applied across all periodontal classes in accordance to systemic antibody levels (separate coding/row): green = low, yellow = mild, orange = moderate, red = high levels.

GI, gingival inflammation index; PPC, Periodontal Profile Class; TL, tooth loss.

a Data adjusted for race/center, age, sex, diabetes, body mass index, smoking (3 levels: current smoker, former smoker, nonsmoker), education (3- levels: basic, intermediate, advanced), and dental utilization (frequent dental visits, episodic dental visits).

## Discussion

This is the first study that characterizes the microbial biofilm burden and the systemic antibody response within periodontal profile classes in a sizable sample of middle-aged Americans. All PPC disease classes presented higher bacterial loads and/or antibody levels compared to PPC-I (health), which may reflect the biological and microbiological imbalances that underlie these different periodontal conditions. The biologically distinct disease subtypes identified may represent homogeneous groups of individuals with similar risk for disease development, progression, and response to treatment and aid in identifying therapies that directly addresses the patient’s needs. Importantly, the PPC system derives from clinical measurements alone and provides biological information without the need for additional microbiological testing, which would facilitate the delivery of dental chairside precision care.

Our study presents both confirmatory and novel findings. In accordance to previous studies (Ebersole et al. 1987), men and African Americans showed high bacteria and systemic antibody responses, which support a biologic basis for their increased risk for periodontal disease. Our results also confirm that classes of health (PPC-I), mild to moderate disease (PPC-II to PPC-III), and severe disease (PPC-IV) demonstrate microbiological shifts of low levels of pathogens to “incipient” dysbiosis (slight increase of gram-negative anaerobes) to a higher level of gram-negative, strictly anaerobic pathogens (Table 3). For these 4 classes (health, mild, moderate, severe), the systemic antibody responses were generally in accordance with the microbial biofilm composition (Table 4). Novel findings in this study include the microbial and antibody characteristics of “hidden” classes: high gingival inflammation (PPC-V), reduced periodontium (PPC-VI), and severe tooth loss (PPC-VII). The classes with a high gingival index (PPC-V) and severe tooth loss (PPC-VII) showed the highest subgingival colonization of orange and red complex bacteria, consistent with a dysbiotic state. From a treatment standpoint, patients in these classes may greatly benefit from combining the clinical findings with the microbiological profiling for a therapeutic intervention focused on preventive treatment regimens that target a microbial burden decrease (increase visit and hygiene frequency, antibiotic treatments). This may contrast with a precise treatment indication for a patient with reduced periodontium (PPC-VI), which has similar periodontal measurements as the high inflammation class (PPC-V) but a health-compatible microbiome. Because of their high risk for tooth loss (Morelli et al. 2018), the treatment selection for reduced periodontium (PPC-VI) patients may include host-modulatory therapies to reduce the exuberant and/or persistent inflammation toward a healthy commensal microbiota.

We found that the PPC classes were also distinct systemically. Hyperinflammatory (PPC-V) individuals showed a moderate to high systemic antibody response against periodontal pathogens. Importantly, individuals in this class present a high heritability (over 50%; Agler et al. 2019), suggesting that this hyperinflammatory trait may have an underlying biological systemic component that also affects other tissues and predisposes for other inflammatory diseases. In support of this, the hyperinflammatory class (PPC-V) had a high systemic microbial response and the second highest stroke incidence rate among all study participants (Beck et al. 2018b). Together, these local and systemic inflammatory findings support PPC-V as a biologically homogeneous, hyperinflammatory group.

Additional “hidden” classes (PPC-VI to PPC-VII) presented opposite microbial and host response profiles. The “moderate tooth loss” class (PPC-VI) showed a low-level periodontal pathogen profile in the biofilm that was similar to health but a general moderate/high systemic IgG antibody response. Because antibodies are generally stable (Lakio et al. 2009), this suggests that these patients likely had a high microbial burden during their lifetime. Contrary, individuals in the “severe tooth loss” class (PPC-VII) were characterized by high levels of biofilm periodontal pathogens but a general low systemic antibody IgG response (acquired immune response), which could suggest that these individuals are not immunocompetent and require further medical treatment to address a systemic underlying cause. The usage of these microbiological findings to improve the process of periodontal treatment care selection requires further investigation.

This study has limitations. Periodontitis is characterized by a complex network of mixed microbial communities that can present species variations based on a several factors, including micron-scale biogeography. Therefore, analysis of 8 periodontal pathogens in 1 sample per individual cannot allow full appreciation of the microbial differences that likely exist between the different periodontal classes. Microbial characterization by high-throughput methods would improve the evaluation of bacterial community interactions (Perez-Chaparro et al. 2014) and could offer new targets for therapeutic interventions. Still, next-generation sequencing studies confirm the role of “orange and red complex” pathogens in the pathobiology of periodontitis, further supporting the value of classic periodontal pathogens in dysbiosis. In addition, because DNA sequencing technologies have not yet led to any changes in periodontal care (Feres et al. 2021), microbiological findings derived from a limited number of periodontal pathogens in a large cohort provide an important foundation for future research to develop.

It is also important to acknowledge that, despite their abundant presence in periodontal lesions, the biological significance of antibody responses in disease pathogenesis is still not clear (Hajishengallis and Korostoff 2017). Periodontitis cases are generally reported to have elevated systemic antibodies relative to health. It has been suggested that the high levels indicate the presence of an effective host response for eliminating the local infection or that there is an inefficient host response (Ebersole et al. 1987; Kinane et al. 1999; Papapanou et al. 2000; Schenkein 2006). High antibody responses have been previously correlated to systemic diseases. The fact that individuals in classes with high gingival inflammation (not necessarily high periodontal destruction) are at higher risk for incident stroke compared to less inflamed periodontal classes (Sen et al. 2018) further supports that the periodontal classes are biologically distinct. Additional studies are needed to clarify the significance of the microbial-host patterns identified.

Finally, prospective validation studies are needed to determine the ability of PPC in identifying biological targets for clinical use. Researchers may find that the use of a data-driven statistical model for identification of distinct periodontal classes may be useful in designing precise biological treatment interventions.

## Author Contributions

J.T. Marchesan, contributed to conception, data analysis, and interpretation, drafted and critically revised the manuscript; K. Moss, contributed to data acquisition, analysis, and interpretation, critically revised the manuscript; T. Morelli, K. Divaris, M. Styner, A.A. Ribeiro, J. Webster-Cyriaque, contributed to data interpretation, critically revised the manuscript; F.R. Teles, contributed to data analysis and interpretation, critically revised the manuscript; J. Beck, contributed to conception, design, data acquisition, and interpretation, critically revised the manuscript. All authors gave final approval and agree to be accountable for all aspects of the work.

## Supplemental Material

sj-pdf-1-jdr-10.1177_00220345211009767 – Supplemental material for Distinct Microbial Signatures between Periodontal Profile Classes Click here for additional data file. Supplemental material, sj-pdf-1-jdr-10.1177_00220345211009767 for Distinct Microbial Signatures between Periodontal Profile Classes by J.T. Marchesan, K. Moss, T. Morelli, F.R. Teles, K. Divaris, M. Styner, A.A. Ribeiro, J. Webster-Cyriaque and J. Beck in Journal of Dental Research
