# Supplementary material for: Distinct Microbial Signatures between Periodontal Profile Classes
Source: J Dent Res. 2021 Apr 27;100(12):1405–13. doi: 10.1177/00220345211009767 (PMC8529299; doi:10.1177/00220345211009767)
Supplement: sj-pdf-1-jdr-10.1177_00220345211009767 – Supplemental material for Distinct Microbial Signatures between Periodontal Profile Classes [file sj-pdf-1-jdr-10.1177_00220345211009767.pdf]

**Supplemental Material**

**Distinct Microbial Signatures Among Periodontal Profile Classes**

Julie T. Marchesan, Kevin Moss, Thiago Morelli, Flavia R. Teles, Kimon Divaris, Styner M, Apoena DeAguiar Ribeiro, Jennifer Webster-Cyriaque, James Beck.

## Methods

### Study population and disease classification

The ARIC study, supported by the National Heart, Lung, and Blood Institute, is a prospective investigation of the etiology and natural history of atherosclerosis and clinical cardiovascular disease in 4 US communities (Jackson, Miss; Washington County, Maryland; suburban Minneapolis, Minn; and Forsyth County, North Carolina). A sample of 15,792 community-dwelling residents aged 45 to 64 years at baseline took part in an evaluation of cardiovascular risk factors and sequelae. The dataset is publicly available upon request to the parent study (https://www.ncbi.nlm.nih.gov/projects/gap/cgi-bin/study.cgi?study_id=phs000280.v6.p1).

The Dental ARIC (DARIC) was an ancillary (supplementary) study funded by the NIDCR conducted at ARIC visit 4 (1996-1998). The DARIC cohort study comprised 6,793 dentate individuals living in the four United States communities described above. Demographics and medical history were recorded. The Dental ARIC consisted of an oral examination, with collection of the following clinical measures: Probing depth (PD) and cemento-enamel junction (CEJ) - determined with a UNC-15 periodontal probe at 6 sites/tooth; bleeding on probing (BOP), assessed after probing each tooth surfaces. Gingival index, Loe & Silness – determined as 0=normal gingiva; 1=mild inflammation: slight change in color, slight edema, no BOP; 2= moderate inflammation: redness, edema, and glazing, BOP. 3=severe inflammation: marked redness and edema, ulceration, tendency to bleed spontaneously. Plaque score - determined

on a 0-3 scale. The dental examiners were calibrated against a standard examiner, as well as each other.

The current study consisted of a subset of 1,450 adult dentate participants of the DARIC study that had biofilm collection and serum collection in addition to the clinical measurements described above. Individuals requiring antibiotic prophylaxis were excluded from the study.

**Collection and analysis of Bacterial biofilm composition and Serum IgG Antibody Titers**

Plaque and serum samples were collected and evaluated according to methods previously described (Beck et al 2005). Briefly, four plaque samples were obtained from the mesial surface of all first molars using a sterile curette. If the tooth was missing, sampling occurred on the mesial surface of the nearest most posterior adjacent tooth. The site was isolated with a cotton roll and removed of gross supragingival plaque or debris if present and gently dried with sterile gauze or air stream. Plaque samples will be taken by placing a sterile curette to the base of each pocket and, with a single stroke, pressing against the tooth at the depth of the pocket, removing a sample for transfer to the storage buffer. Supragingival biofilm was not collected. The biofilm sample was placed into a tube containing 0.1 mL sterile TE buffer. Following sample collection, 0.1 mL of 0.5 M NaOH (sterile) was added using the plunger-style dispenser, mixed well, and stored at −80◦C for further analysis. In the current study, one biofilm sample from each individual was assayed by DNA-DNA checkerboard for the eight periodontal pathogens. Total counts reflect a sum of these targeted pathogens for each individual. Serum samples were collected and frozen at −80◦C until further analysis. The samples were assayed for IgG antibody levels using the checkerboard immunoblotting technique described by Beck et al. 2005.

**Supplemental Table 1**. Periodontal Profile Class definitions according to different references.

| Beck et al 2020 | | Morelli et al 2017 | |
|---|---|---|---|
| Stage | Description | Class | Description |
| I | Health | A | Health |
| II | Mild | B | Mild Disease |
| III | Moderate | E | Posterior Disease |
| IV | Severe Disease | G | Severe Disease |
| V | Mild Tooth Loss (TL)/High Gingival Index (GI) | C | High gingival index (GI) scores |
| VI | Moderate (Mod) Tooth Loss (TL)/Reduced (Red) Periodontium (Perio) | D | Tooth Loss |
| VII | Severe Tooth Loss (TL) | F | Lower Incisors |

**Supplemental Table 2**: Number of subjects by Periodontal Profile Classes (PPC) and World Workshop 2017 (WW17 modified*) periodontal disease classification.

| | WW17 Stage I | WW17 Stage II | WW17 Stage III | WW17 Stage IV | Total (PPC) |
|---|---|---|---|---|---|
| PPC-Stage I (Health) | 34 | 265 | 72 | 13 | 384 |
| PPC-Stage II (Mild) | 10 | 141 | 74 | 10 | 235 |
| PPC-Stage III (Moderate) | 0 | 34 | 163 | 19 | 216 |
| PPC-Stage IV (Severe) | 0 | 13 | 90 | 20 | 123 |
| PPC-Stage V (Mild TL/High GI) | 0 | 30 | 23 | 70 | 123 |
| PPC-Stage VI (Red Perio) | 1 | 16 | 17 | 140 | 174 |
| PPC-Stage VII (Severe TL) | 0 | 0 | 0 | 195 | 195 |
| Total (WW17) | 45 | 499 | 439 | 467 | 1450 |

*The clinical data used to model the World Workshop grades does not contain longitudinal measures of clinical attachment loss or radiographic bone loss; the stages were defined by clinical attachment levels and defined as a modified version, described previously (Beck et al. 2019).
TL= tooth loss; GI= gingival index.

**Supplemental Table 3A**. Mean (se) adjusted* log microbial counts by the Centers for Disease Control/American Academy of Periodontology definition of disease.

| Bacterial biofilm | Health n=158 | Mild n=431 | Moderate n=588 | Severe n=273 | p-value |
|---|---|---|---|---|---|
| *P. intermedia* | 2.87 (0.16) | 2.82 (0.10) | 2.70 (0.08) | 2.78 (0.13) | 1.00 |
| *P. nigrescens* | 2.69 (0.15) | 2.76 (0.09) | 2.89 (0.08) | 2.97 (0.12) | 1.00 |
| *F. nucleatum* | 2.91 (0.16) | 2.98 (0.10) | 3.06 (0.08) | 3.04 (0.13) | 1.00 |
| *C. rectus* | 2.80 (0.14) | 2.75 (0.08) | 2.91 (0.07) | 2.96 (0.11) | 1.00 |
| *P. gingivalis* | 2.19 (0.13) | 2.32 (0.08) | 2.40 (0.07) | 2.68 (0.10) | 0.11 |
| *T. forsythia* | 2.70 (0.13) | 2.41 (0.08) | 2.55 (0.07) | 2.79 (0.10) | 0.17 |
| *T. denticola* | 2.74 (0.14) | 2.66 (0.08) | 2.73 (0.07) | 2.84 (0.11) | 1.00 |
| *A. actinomycetemcomitar* | 2.73 (0.13) | 2.65 (0.08) | 2.78 (0.06) | 2.74 (0.10) | 1.00 |

**Supplemental Table 3B**. Mean (se) adjusted* log microbial counts by World Workshop 2017 definition of disease (modified)**.

| Bacterial biofilm | Stage I N=45 | Stage II N=499 | Stage III N=439 | Stage IV N=467 | p-value |
|---|---|---|---|---|---|
| *P. intermedia* | 2.67 (0.30) | 2.86 (0.09) | 2.61 (0.10) | 2.83 (0.10) | 1.00 |
| *P. nigrescens* | 2.34 (0.29) | 2.91 (0.09) | 2.83 (0.09) | 2.85 (0.10) | 1.00 |
| *F. nucleatum* | 1.94 (0.30) | 3.14 (0.09) | 3.02 (0.10) | 2.98 (0.10) | 0.02 |
| *C. rectus* | 2.67 (0.25) | 2.80 (0.08) | 2.83 (0.08) | 2.96 (0.08) | 1.00 |
| *P. gingivalis* | 1.61 (0.24) | 2.44 (0.08) | 2.43 (0.08) | 2.42 (0.08) | 0.09 |
| *T. forsythia* | 2.45 (0.24) | 2.45 (0.08) | 2.57 (0.08) | 2.70 (0.08) | 1.00 |
| *T. denticola* | 2.59 (0.26) | 2.72 (0.08) | 2.72 (0.08) | 2.77 (0.09) | 1.00 |
| *A. actinomycetemcomitans* | 2.21 (0.23) | 2.65 (0.07) | 2.77 (0.08) | 2.82 (0.08) | 0.57 |

p-values based upon general linear models. ***Data adjusted for race/center, age, sex, diabetes, body mass index, smoking (3-levels:current smoker, former smoker, non-smoker), education (3-levels: basic, intermediate, advanced) and dental utilization (frequent dental visits, episodic dental visits). p-values based on general linear models.

** The clinical data used to model the World Workshop grades does not contain longitudinal measures of clinical attachment loss or radiographic bone loss; the stages were defined by clinical attachment levels and defined as a modified version, described previously (Beck et al. 2019).

Color-coding system applied across all periodontal classes in accordance to levels of microoganisms (separate coding/row): green=low, yellow=mild, orange=moderate, red=high levels.

**Supplemental Table 4A.** Mean (se) adjusted* log microbial IgG levels stratified by the Centers for Disease Control/American Academy of Periodontology definition of disease (n=1,450).

| | Health | Mild | Moderate | Severe | p-value |
|---|---|---|---|---|---|
| ***P. intermedia*** | 1.38 (0.06) | 1.38 (0.03) | **1.52 (0.03)** | **1.59 (0.04)** | **0.004** |
| *P. nigrescens* | 1.73 (0.05) | 1.73 (0.03) | 1.78 (0.03) | 1.76 (0.04) | 1.00 |
| *F. nucleatum* | 0.85 (0.05) | 0.85 (0.03) | 0.85 (0.03) | 0.82 (0.04) | 1.00 |
| ***C. rectus*** | 1.04 (0.05) | 1.03 (0.03) | **1.16 (0.03)** | **1.21 (0.04)** | **0.009** |
| ***P. gingivalis*** | 0.98 (0.06) | 0.97 (0.03) | **1.23 (0.03)** | **1.52 (0.04)** | **<0.0001** |
| *T. forsythia* | 1.29 (0.05) | 1.26 (0.03) | 1.33 (0.02) | 1.35 (0.04) | 1.00 |
| *T. denticola* | 1.30 (0.04) | 1.28 (0.02) | 1.35 (0.02) | 1.35 (0.03) | 0.63 |
| *A. actinomycetemcomitans* | 1.76 (0.05) | 1.78 (0.03) | **1.87 (0.02)** | **1.91 (0.04)** | 0.10 |

**Supplemental Table 4B.** Mean (se) adjusted* log microbial IgG levels stratified by WW17 periodontal disease classification (n=1,450).

| | Stage I | Stage II | Stage III | Stage IV | p-value |
|---|---|---|---|---|---|
| *P. intermedia* | 1.39 (0.10) | 1.44 (0.03) | 1.56 (0.03) | 1.45 (0.03) | 0.24 |
| *P. nigrescens* | 1.82 (0.10) | 1.75 (0.03) | 1.80 (0.03) | 1.72 (0.03) | 1.00 |
| *F. nucleatum* | 0.85 (0.10) | 0.83 (0.03) | 0.85 (0.03) | 0.85 (0.03) | 1.00 |
| *C. rectus* | 1.06 (0.10) | 1.06 (0.03) | 1.20 (0.03) | 1.10 (0.03) | 0.09 |
| ***P. gingivalis*** | 0.95 (0.11) | 1.00 (0.03) | **1.36 (0.03)** | **1.22 (0.04)** | **<0.0001** |
| *T. forsythia* | 1.36 (0.09) | 1.30 (0.03) | 1.33 (0.03) | 1.29 (0.03) | 1.00 |
| *T. denticola* | 1.29 (0.07) | 1.31 (0.02) | 1.36 (0.02) | 1.31 (0.02) | 1.00 |
| *A. actinomycetemcomitans* | 1.82 (0.09) | 1.81 (0.03) | 1.90 (0.03) | 1.82 (0.03) | 0.94 |

p-values based upon general linear models. Bold indicates significant using PPC-Stage I as reference (Bonferroni corrected). ***Data adjusted for race/center, age, sex, diabetes, body mass index, smoking (3-levels: current smoker, former smoker, non-smoker), education (3-levels: basic, intermediate, advanced) and dental utilization (frequent dental visits, episodic dental visits). p-values based upon general linear models.

Color-coding system applied across all periodontal classes in accordance to systemic antibody levels (separate coding/row): green=low, yellow=mild, orange=moderate, red=high levels.
